# Transcriptomic insights on the ABC transporter gene family in the salmon louse *Caligus rogercresseyi*

**DOI:** 10.1186/s13071-015-0801-x

**Published:** 2015-04-09

**Authors:** Valentina Valenzuela-Muñoz, Armin Sturm, Cristian Gallardo-Escárate

**Affiliations:** Laboratory of Biotechnology and Aquatic Genomics, Interdisciplinary Center for Aquaculture Research (INCAR), University of Concepción, PO. Box 160-C, Concepción, Chile; Institute of Aquaculture, University of Stirling, Stirling, FK9 4LA Scotland UK

**Keywords:** *Caligus rogercresseyi*, ABC transporters, RNA-Seq, Deltamethrin, Azamethiphos, SNPs

## Abstract

**Background:**

ATP-binding cassette (ABC) protein family encode for membrane proteins involved in the transport of various biomolecules through the cellular membrane. These proteins have been identified in all taxa and present important physiological functions, including the process of insecticide detoxification in arthropods. For that reason the ectoparasite *Caligus rogercresseyi* represents a model species for understanding the molecular underpinnings involved in insecticide drug resistance.

**Methods:**

llumina sequencing was performed using sea lice exposed to 2 and 3 ppb of deltamethrin and azamethiphos. Contigs obtained from *de novo* assembly were annotated by Blastx. RNA-Seq analysis was performed and validated by qPCR analysis.

**Results:**

From the transcriptome database of *C. rogercresseyi*, 57 putative members of ABC protein sequences were identified and phylogenetically classified into the eight subfamilies described for ABC transporters in arthropods. Transcriptomic profiles for ABC proteins subfamilies were evaluated throughout *C. rogercresseyi* development. Moreover, RNA-Seq analysis was performed for adult male and female salmon lice exposed to the delousing drugs azamethiphos and deltamethrin. High transcript levels of the ABCB and ABCC subfamilies were evidenced. Furthermore, SNPs mining was carried out for the ABC proteins sequences, revealing pivotal genomic information.

**Conclusions:**

The present study gives a comprehensive transcriptome analysis of ABC proteins from *C. rogercresseyi,* providing relevant information about transporter roles during ontogeny and in relation to delousing drug responses in salmon lice. This genomic information represents a valuable tool for pest management in the Chilean salmon aquaculture industry.

**Electronic supplementary material:**

The online version of this article (doi:10.1186/s13071-015-0801-x) contains supplementary material, which is available to authorized users.

## Background

*Caligus rogercresseyi*, a sea louse, is a widely prevalent parasite in the Chilean aquaculture industry [1,2]. This ectoparasite belongs to the Caligidae family, which includes species such as *Lernaeocera branchialis*, *Caligus clemensi*, and *Lepeophtheirus salmonis*, with this final species having greater prevalence in countries such as Scotland, Norway, Canada, and England [3]. Although not associated with host mortality, an infection of *C. rogercresseyi* is a highly stressful condition for fish, which is reflected by lower culture performance and a depression of the host’s immune system, which thus increases susceptibility to other types of contagious diseases [1-4]*.*

Numerous delousing drugs have been used worldwide for the control of this ectoparasite [5]. However, many studies suggest that the effectiveness of different treatments for sea lice principally depends on the developmental stage of the parasite and the delousing drugs used, for example, there are reports that have mentioned the effect of emamectin benzoate in chitin synthesis which results in a fragile exoskeleton after moulting [5-9]. One of the protein families which has shown detoxifying effects against drugs are the ABC transporters. These types of transporters have been widely related to the generation of resistance to emamectin benzoate in *L. salmonis* [10,11].

The ATP-binding cassette (ABC) proteins family is ubiquitous in the animal kingdom, and has even been found in plants. Most of these ABC proteins are integral membrane proteins that use ATP to transport biomolecules through the plasma membrane [12]. In eukaryotes, this family has a characteristic organization marked by two transmembrane domains (TMD) that are formed by five or six helixes which determine the specificity of the transporter [13,14]. Moreover, this family also presents two cytosolic nucleotide binding domains (NBD), and these bind and hydrolyze the ATP necessary for transporting substances across the membrane [13,14]. The NBD is a highly conserved sequence that presents characteristic sites such as Q-look, the H-motif, and the LSGGQ-motif [14,15]. It is possible to divide ABC transporters into the following two groups: those present only in prokaryotes and which require substrate-binding proteins for transport, and those found only in eukaryotes and that bind directly to the substrate from the interior of the cell [15]. The substances transported by ABC transporters include amino acids, sugars, lipids, inorganic ions, polysaccharides, metals, peptides, and toxic substances [16].

The ABC proteins family is comprised of subfamilies that are differentiated according to domain and sequence structures [16]. In mammals, seven subfamilies (A-G) have been identified, whereas in arthropods and zebra fish, eight subfamilies (A-H) have been found [12,16]. While in prokaryotic organisms such as *Escherichia coli,* the ABC family has been subdivided into 22 subfamilies with transporter activity and 24 with exporter activity [17]. Of the eight subfamilies describe in arthropod, the E and F subfamilies are the only ones without transporter functions. The ABC transporter E subfamily (ABCE) members act as inhibitors of RNase L and participate in assembling the preinitiation complex, while the ABCF subfamily plays a role in assembling ribosomes and in protein translation [12]. Currently, the ABC transporter subfamilies have only been characterized in eight arthropod species, which are *D. melanogaster* [13]*, Anopheles gambiae* [18], *Apis mellifera* [19], *Bombix mori* [20]*, Tribolium casteneum* [21], *Tetranychus urticae* [22]*, Daphia pulex* [12]*,* and, recently, *Tigriopus japonicus* [23].

The ABCB subfamily is especially of interest given its ability to transport drugs [24,25], with P-glycoprotein (P-gp) being the first transporter identified within this family [26]. Furthermore, the ABCC and ABCG families have been reported to have a similar detoxifying function [16,27]. Given their roles related to the detoxification of drugs, these proteins have been termed multidrug resistance proteins (MRPs). In invertebrates, MRPs have been associated with the generation of resistance to insecticides, including in species such as *Caenorhabditis elegans*, *Tricho plusiani* [28], and *Aedes aegypti* [29], among others. In the ectoparasites *Lepeophtheirus salmonis* [10] and *Caligus rogercresseyi* [30], a close association has been found between the generation of resistance to emamectin benzoate (EMB) and the transcriptomic response of P-gp. Likewise, an exhaustive study on the different ABC transporter families in *D. pulex* was carried out with the purpose of understanding the adaptation mechanisms that this crustacean uses in response to toxic compounds [12], and a recent study in *L. salmonis* characterized an additional four MRPs [31]. However, these MRPs in *L. salmonis* did not present differences in transcript expression between EMB resistant/susceptible strains.

*C. rogercresseyi* is an ectoparasite responsible for significant economic losses in the Chilean salmon aquaculture industry, and, as with *L. salmonis*, this species has demonstrated resistance to the drugs currently used in infestation control [32-34]. Given the role that ABC transporters play in pharmaceutical resistance in invertebrates, the objective of the present study was to identify members of the distinct ABC subfamilies and determine expression patterns during the distinct stages of development in *C. rogercresseyi*. Moreover, RNA-Seq analysis was performed in adult salmon lice exposed to the delousing drugs deltamethrin and azamethiphos in order to determine the modulation of distinct ABC proteins in response to drugs currently used in the control of *C. rogercresseyi*.

## Methods

### Samples and bioassays

Adult male and female sea lice were collected from a commercial farm located in Region de los Lagos of Chile (41°40′48.5″S; 73°02′31.34″O¨). Permissions for sea lice collection were authorized by Marine Harvest S.A, Ruta 226, Km. 8, Camino El Tepual, Puerto Montt, Chile.

For the bioassays, deltamethrin (AlphaMax®) was prepared via serial dilutions with seawater to four concentrations (0, 1, 2, 3 ppb). A stock solution of 10 ppm was also prepared for each bioassay by diluting 1 ml of deltamethrin in 999 ml of seawater. In regards to azamethiphos (Bayer®), a stock solution of 1 ppm diluted in methanol and three serial dilutions with seawater to four concentrations (0, 1, 3, 10 ppb) were prepared. Ten sea lice adults (five females and five males) were exposed to each concentration of deltamethrin and azamethiphos using petri plates containing 50 ml of seawater (total individuals = 30). Each experiment was carried out in triplicate. The exposure period to either deltamethrin or azamethiphos was 40 and 30 min, respectively. During exposure, salmon lice were maintained at 12°C. After 24 h, the organisms were fixed in theRNAlater® RNA Stabilization Reagent (Ambion, USA) and stored at −80°C for subsequent RNA extraction. The protocols for bioassays were performed according to the SEACH Consortium (2006). All laboratory infections and culture procedures were carried out under guidelines approved by the ethics committee of the University of Concepción and under appropriate veterinary supervision.

### High-throughput sequencing

The concentration 2 ppb and 3 ppb of deltamethrin or azamethiphos, respectively, was determined as EC50 in the bioassay. For this reason 15 females and 15 males of each group exposed to these concentrations, were used for MiSeq cDNA libraries preparation. Total RNA was extracted from pooled individuals for each sex (N = 10) using the RiboPure™ Kit (Ambion®, Life Technologies™, USA) following the manufacturer’s instructions. Quantity, purity, and quality of isolated RNA were measured in the TapeStation 2200 (Agilent Technologies Inc., Santa Clara, CA, USA) using the R6K Reagent Kit according to the manufacturer’s instructions; samples with RIN over 8.0 were used for library preparation. Subsequently, double-stranded cDNA libraries were constructed using the TruSeq RNA Sample Preparation Kit v2 (Illumina®, San Diego, CA, USA). Two biological replicates for each sample pool were sequenced by the MiSeq (Illumina®) platform using sequenced runs of 2x251 paired-end reads at the Laboratory of Biotechnology and Aquatic Genomics, Interdisciplinary Center for Aquaculture Research (INCAR), University of Concepción, Chile. The cleaned short read sequences were deposited in the Sequence Read Archive (SRA) (http://www.ncbi.nlm.nih.gov/sra) under the accession number SRX864101 and SRX864102 for deltamethrin and azamethiphos, respectively.

### Sequence annotation and RNA-Seq analysis

From the EST-database generated for *C. rogercresseyi* [35], contigs were annotated using a database constructed from ABC transporter sequences described for *D. pulex* [12] and enriched with EST data for arthropods in order to determine putative gene descriptions. A cutoff E-value of 1E-05 was used.

The same EST database generated for *C. rogercresseyi* was used as a reference for RNA-Seq analysis. Using the CLC Genomic Workbench software, the reads obtained from female and male adult controls and individuals exposed to azamethiphos or deltamethrin were separately mapped against ABC transporter contigs. The RNA-Seq settings were a minimum length fraction = 0.6 and a minimum similarity fraction (long reads) = 0.5. The expression value was set as a reads per kilobase of exon model (RPKM). This normalized the number of reads to the size of assembled contigs and allowed for assessing the transcripts that were overexpressed among different groups. Furthermore, to compare differentiated transcript responses between sexes, the reads obtained for females and males were mapped separately over the ABC contigs using the RNA-Seq settings previously described [35]. A similar analysis was carried out between control and exposure groups using the new contigs obtained from *de novo* assembly as a reference. The metric distance was calculated using the Manhattan method, where the mean expression level in 5–6 rounds of k-means clustering was subtracted. Finally, a Kal’s statistical analysis test [36] was used to compare gene expression levels for larval stages and adults in terms of the log2 fold change (P = 0.0005; FDR corrected).

### Amino acid sequence analyses

Protein alignments were conducted using MUSCLE, and phylogenetic trees were constructed using the neighbor-joining method with 1,000 bootstrap repetitions. Both analyses were carried out in Geneious 6.0.5 [37].

### qPCR validation

Contig sequences of ABC subfamilies were obtained from the Illumina MiSeq database for *C. rogercresseyi* and used as a template for primer design with the Primer3 Tool [38] included in the Geneious Pro software [37] (Additional file 1: Table S1). For gene amplification, total RNA was isolated from sea louse exposed to 3 ppb of azamethiphos and 2 ppb of deltamethrin, using the TRI Reagent (Invitrogen, Carlsbad, CA, USA) protocol. The purity was determined (ratio A260/A280) with a Nanodrop ND1000 spectrophotometer (Thermo Fisher Scientific, Copenhagen, USA), and the integrity was determined by agarose gel under denaturant conditions. From 200 ng/μl of total RNA, cDNA was synthetized using the RevertAid H Minus First Strand cDNA Synthesis Kit (Thermo Scientific, Glen Burnie, Maryland, USA). The qPCR runs were performed with StepOnePlus™ (Applied Biosystems, Life Technologies, USA) using the comparative ΔCt method. *β-tubulin* was selected as the housekeeping gene (HKG) [39]*.* Each reaction was conducted with a volume of 10 μL using the Maxima® SYBR Green/ROX qPCR Master Mix (Thermo Scientific, USA). The amplification conditions were as follows: 95°C for 10 min, 40 cycles at 95°C for 30 s, 60°C for 30 s, and 72°C for 30 s. The data obtained were analyzed through the Kruskal-Wallis test with the Statistica software (Version 7.0, StatSoft, Inc.). Statistically significant differences were accepted with a p < 0.05.

### SNPs mining and validation

Using the assembly obtained for all identified ABC transporters, SNPs mining was performed using the Genomics Workbench 5.0.1 software (CLC bio, Denmark). The parameters used were as follows: window length = 11, maximum gap and mismatch count = 2, minimum average quality of surrounding bases = 15, minimum quality of central base = 20, maximum coverage = 100, minimum coverage = 8, minimum variant frequency (%) = 35.0, and maximum expected variations (ploidy) = 2.

## Results

### Identification of ABC transporter subfamily genes from *C. rogercresseyi*

**Figure 1:**
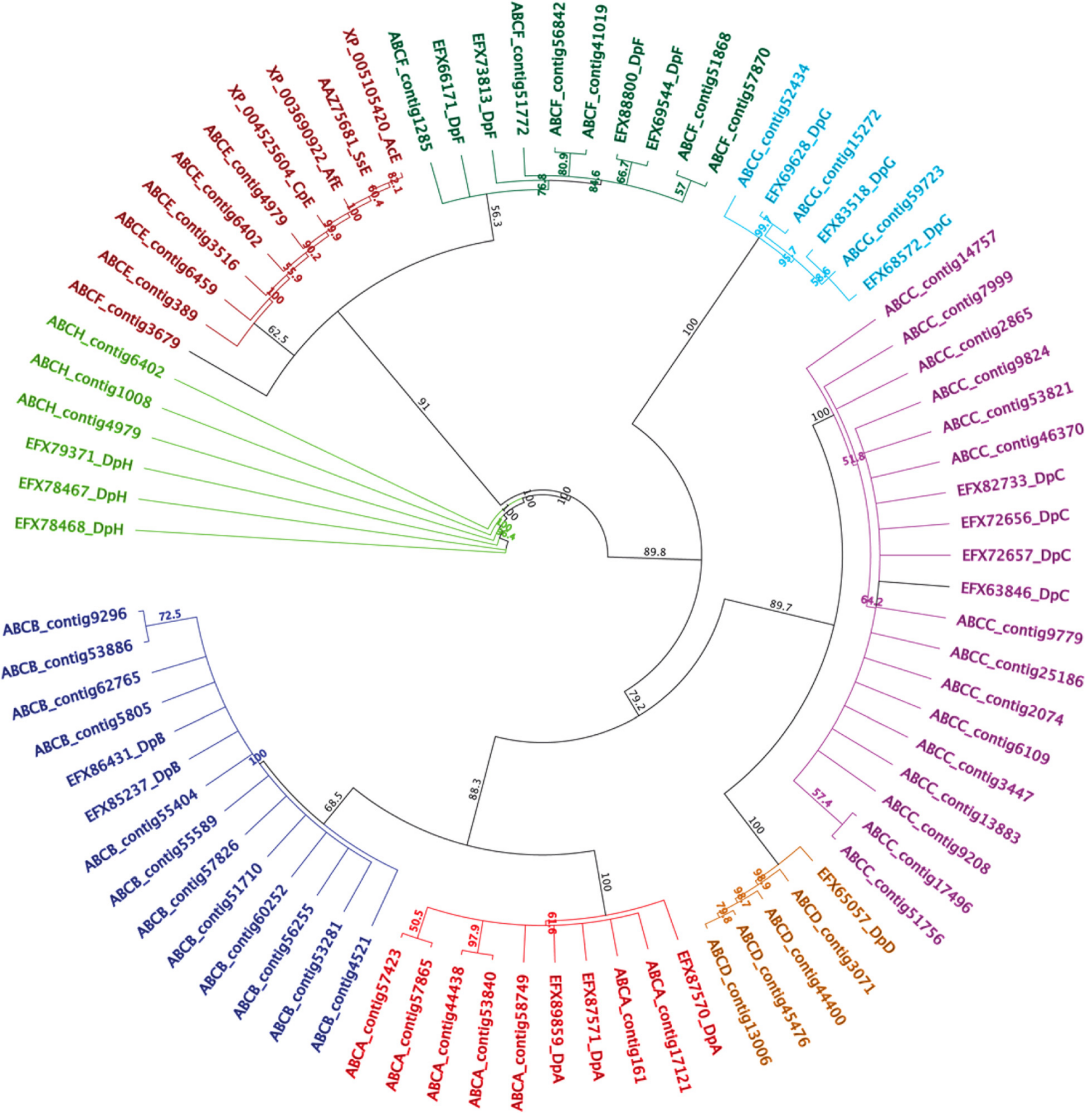
**Phylogenetic relationships between ABC transporter subfamilies identified for** ***C. rogercresseyi*** **.** The number at each node indicates the percentage of bootstrapping after 1,000 replications.

Through BLASTx analysis, 57 full or partial sequences were identified ABC transporter from transcriptome database described by Gallardo-Escárate *et al*. [35] and by using arthropod ABC transporter sequences described in public databases as a reference (Additional file 1: Table S2). Phylogenetic analysis revealed a relationship between seven *C. rogercresseyi* contigs and the ABCA subfamily described in *D. pulex* (Figure 1). From the seven contigs annotated for the ABCA subfamily, one of these presented an encoding sequence of 2,208 amino acids and the structure typical of this subfamily, with a large extracellular loop between the first two helices of the TMD (Table 1). BLASTp analysis demonstrated an identity of 39.8% and 39.3% with sequences described for *Lottia gigantea* (ESP01294) and *Danio rerio* (XP_005173137), respectively. Moreover, twelve contigs were annotated for the ABCB subfamily. Phylogenetic analysis grouped these sequences with the ABCA subfamily described for *D. pulex* (Figure 1)*.* Contig 4,521 appeared as a full transporter encoding for 2,064 amino acids (Table 1). This also presented a 100% identity to the P-gp previously described in *L. salmonis* (ADT63773) and *C. rogercresseyi* (AHC54388). Thirteen contigs presented homology with the ABCC subfamily (Figure 1), with nine presenting high homology to ABCC1 transporters and four presenting homology with the ABCC4 subfamily (Additional file 1: Table S1). Also, two contigs presented high homology with sulfonylurea receptor (SUR) gene, member of ABCC subfamily (Additional file 1: Table S2). For the ABCD subfamily, four contigs were identified. Phylogenetic analysis grouped these sequences with orthologs described in *D. pulex* (Figure 1). BLASTp analysis demonstrated a 38.1% and 38.2% identity to ABCD4 described in *Homo sapiens* (NP_005041) and *Mus musculus* (NP_033018), respectively. From phylogenetic analysis, a relationship was observed between the ABCE and ABCF subfamilies of *C. rogercresseyi* (Figure 1). The sequences that were annotated to the ABCE subfamily presented an identity of 80.8% and 81.1% with homologs described in *A. florea* (XP_003690992) and *Pediculus humanus* (XP_002424051), respectively. The sequenced members of the ABCF subfamily presented an identity of 55.3% and 55.1% to sequences reported in *A. aegypti* (XP_001654470) and *D. pulex* (EFX69544), respectively. Furthermore, three contigs presented high homology with the ABCG subfamily (Additional file 1: Table S2) and were grouped with members of the ABCG subfamily described for *D. pulex* (Figure 1). Among these, contig 15272 encoded for haft transporters with the NBD-TMD domain traits (Table 1). Finally, three contigs of *C. rogercresseyi* were annotated as ABCH transporters (Figure 1). One of these encoded for 1,045 amino acids and presented an organization similar to that observed for ABCG transporters (Table 1).

**Table 1:** **Amino acid position of conserved domain for ABC subfamily of** ***C. rogercresseyi***

				**Amino acid position**			
**Contig**	**Description**	**Type transporter**	**Lenght predict amino acid sequence**	**TMD1**	**NBD1**	**TMD2**	**NBD2**
Contig 161	ABC protein, subfamily ABCA	Full	2,209	550-754	814-1,032	1,583-1,739	1,885-2,091
Contig 4521	ABC protein, subfamily ABCB	Full	1,131	28-255	336-678	711-988	1,035-1,269
Contig 2865	ABC protein, subfamily ABCC	Full	1,456	284-565	607-818	890-1,168	1,207-1434
Contig 3071	ABC protein, subfamily ABCD	Half	538	7-243	327-532	-	-
Contig 3516	ABC protein, subfamily ABCE	Half	616	-	92-347	-	363-608
Contig 1258	ABC protein, subfamily ABCF	Half	544	-	6-224	-	319-513
Contig15272	ABC protein, subfamily ABCG	Half	975	-	58-238	327-583	-
Contig 6402	ABC protein, subfamily ABCH	Half	745	-	34-249	496-710	-

TMDs: transmembrane domains. NBDs: nucleotide-binding domains.

### Transcriptomic profile of ABC transporters during larval stages and in adult *C. rogercresseyi*

**Figure 2:**
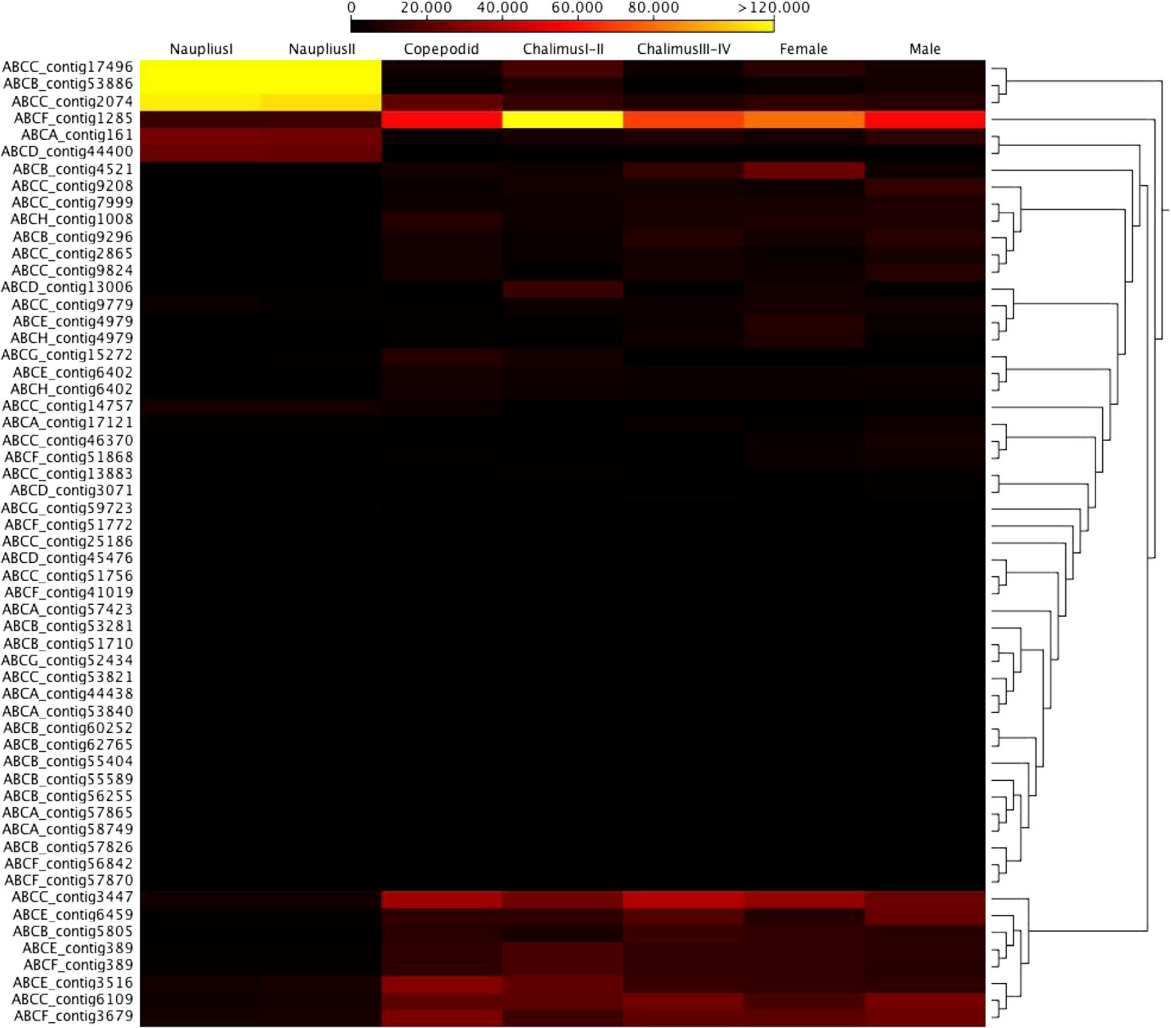
**Heat map generated for ABC transporter families identified from** ***C. rogercresseyi*** **transcriptome data reflecting gene expression values for all developmental stages.**

RNA-Seq analysis was carried out in order to evaluate the transcriptome profiles of ABC transporters during the developmental stages of *C. rogercresseyi* (Figure 2). From the expression profiles in the larval nauplius I-II stages, the subfamilies ABCA/B/C/D were highly regulated. In the infective copepodid stage, the ABCC/E/F/G/H transporters were upregulated. The ABCD/E/F subfamilies were highly regulated in chalimus I-II stages, while in the chalimus III-IV stages, the ABCA/B/C/F subfamilies were upregulated (Figure 2). Furthermore, differences in expression profiles were observed between female and male *C. rogercresseyi* individuals. The ABCD/F subfamilies were upregulated in females, while in males, the ABCB/C subfamilies evidenced high regulation (Figure 2).

### Transcriptomic profiles of ABC transporters in response to delousing drugs

RNA samples obtained from female and male *C. rogercresseyi* individuals exposed to 2 ppb of azamethiphos or 3 ppb of deltamethrin were sequenced using the MiSeq Illumina platform (Table 2). The sequencing runs of salmon lice exposed to azamethiphos yielded a total of 9.06 million reads for males and 10.4 million reads for females, with an average length of 223 and 254 bp, respectively. From adults exposed to deltamethrin, a total of 51.08 M and 27.54 M for males and females were generated, respectively, both with an average length of 219 bp.

**Table 2:** **Statistical summary of** ***Caligus rogercresseyi*** **transcriptome following exposure to Azamethiphos or Deltamethrin**

	**Control**		**Azamethiphos**		**Deltamethrin**	
	**Male**	**Female**	**Male**	**Female**	**Male**	**Female**
Reads (M)	32.29	30.19	9.06	10.40	51.08	27.54
Average length (bp)	142	148	223	254	218	219
Nucleotide number (Gb)	4.59	4.47	2.03	2.64	11.16	6.05
Contigs	38,177	32,172	38,045	40,58	38,536	30,212
Average length (bp)	729	799	785	818	877	922

**Figure 3:**
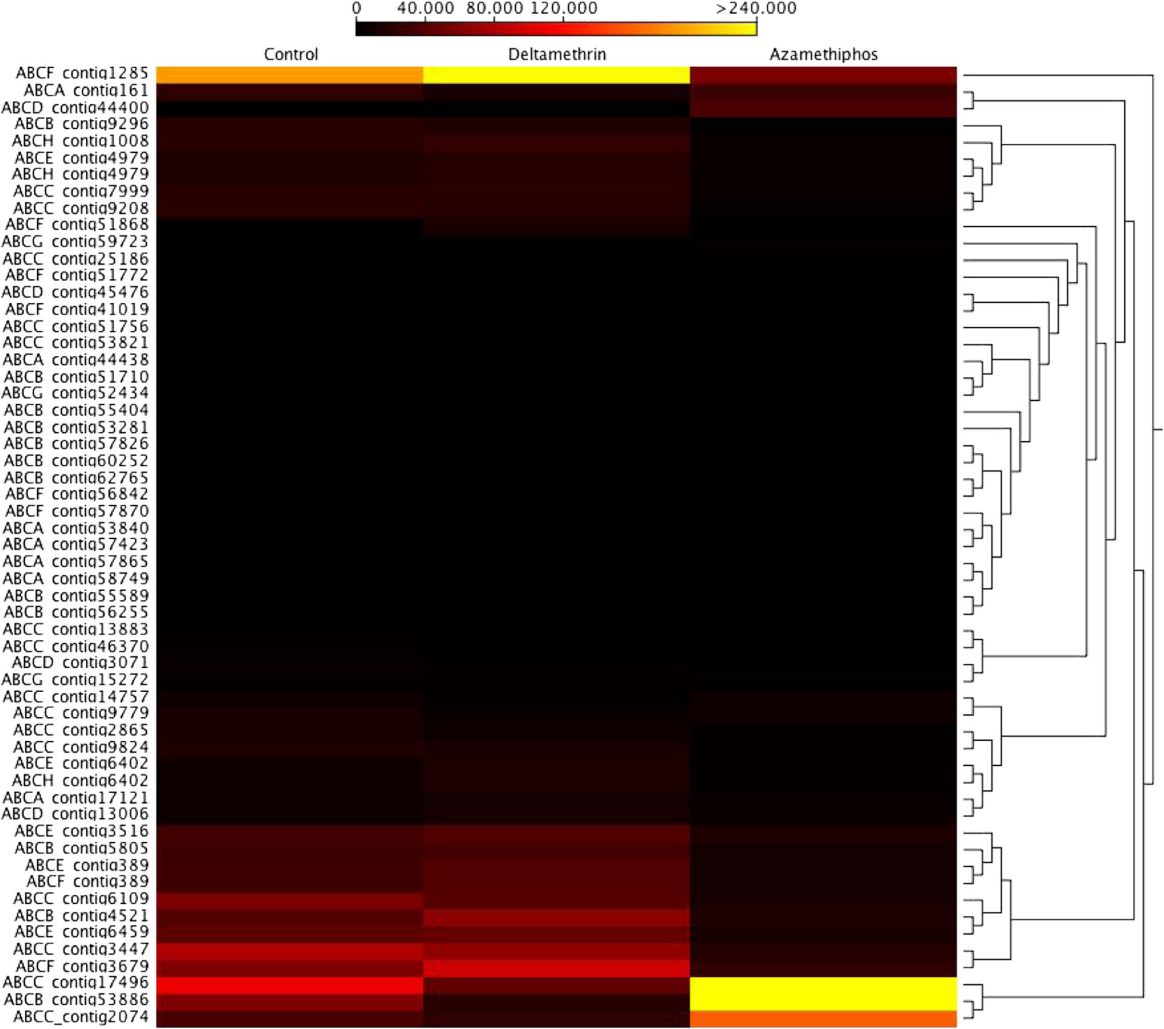
**Cluster of gene expression levels between adult controls, adults exposed to 3 ppb of azamethiphos, and adults exposed to 2 ppb of deltamethrin.**

**Figure 4:**
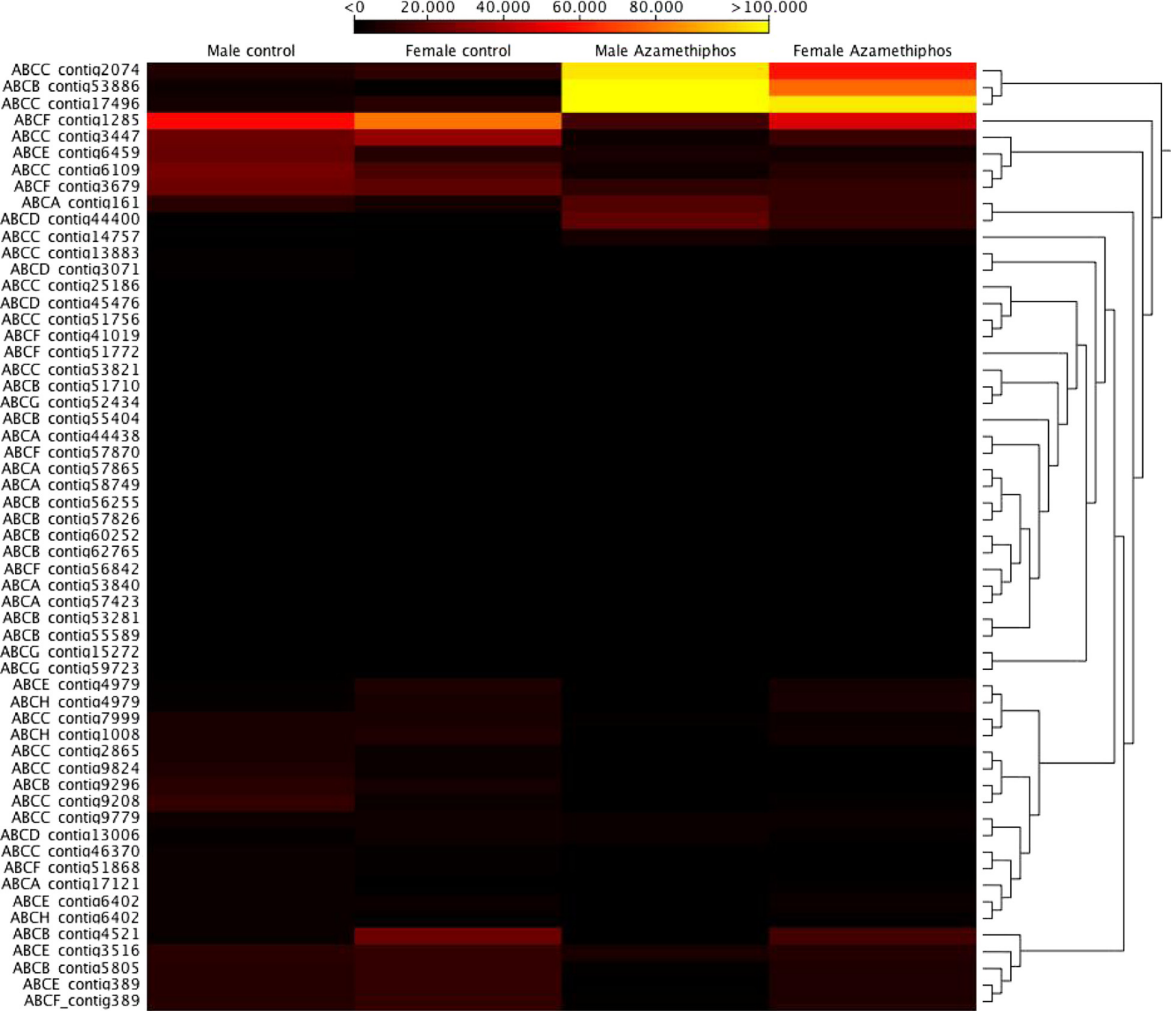
**Cluster of gene expression levels by sex between adult controls and adults exposed to 3 ppb of azamethiphos.**

**Figure 5:**
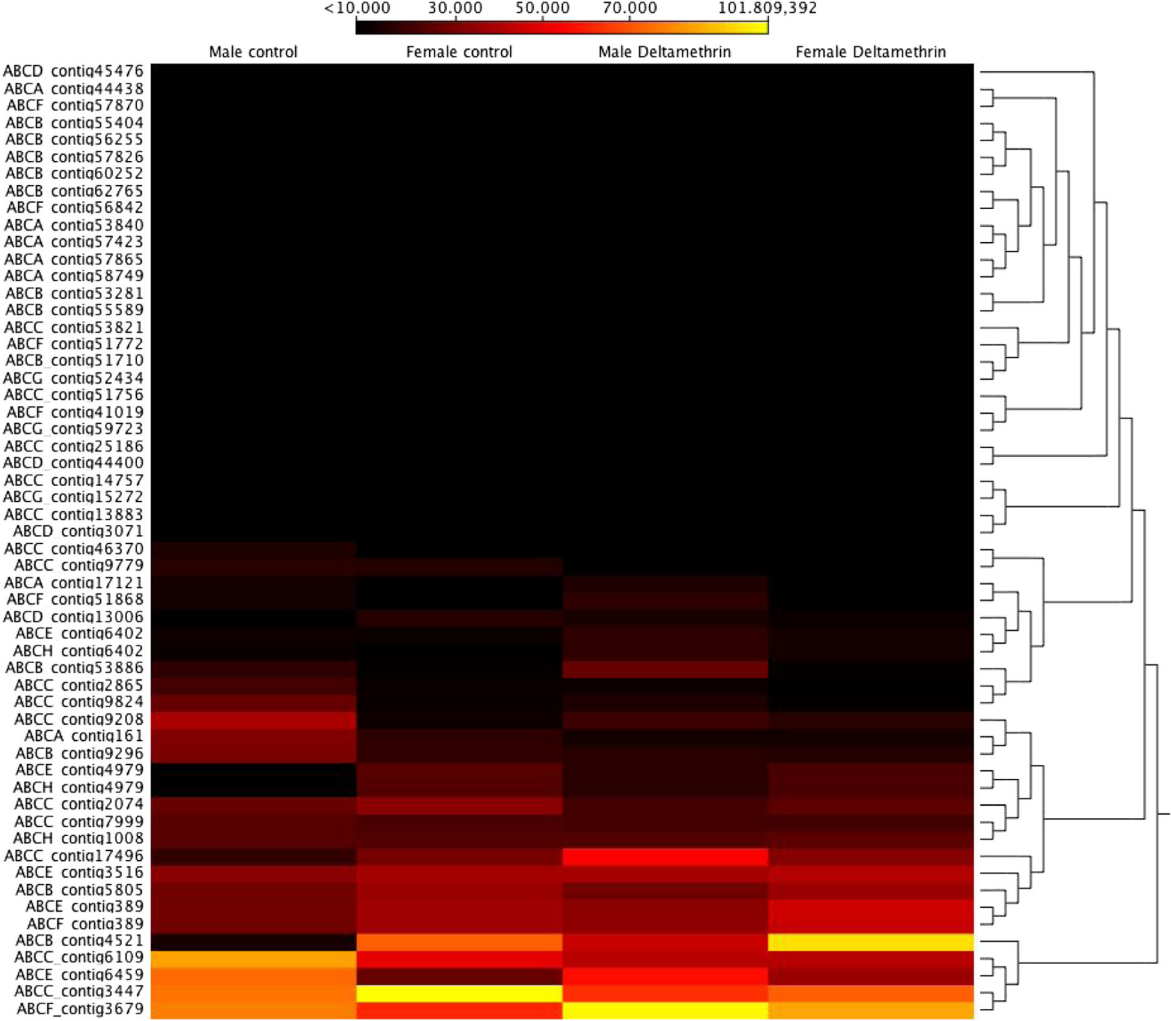
**Cluster of gene expression levels by sex between adult controls and adults exposed to 2 ppb of deltamethrin.**

**Figure 6:**
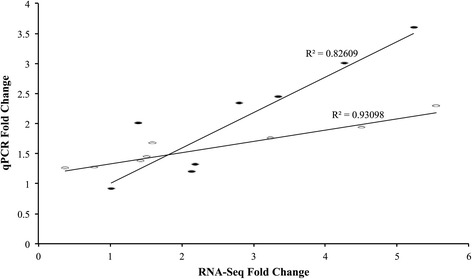
**Transcription expression validation for ABC transporters identified from** ***C. rogercresseyi*** **transcriptome in response to delousing drugs.** Black circle: linear correlation among ABC transporters selected from RNA-seq and qPCR analysis after 3 ppb of azamethiphos exposure on sea lice. White circle: linear correlation among ABC transporters selected from RNA-seq and qPCR analysis after 2 ppb of azamethiphos exposure on sea lice.

Comparing the transcriptomic response of *C. rogercresseyi* adults exposed to azamethiphos, deltamethrin and the control group, the adults exposed to deltamethrin and the control group presented similar expression profiles of ABC transporters (Figure 3). The ABCB and ABCC subfamilies were upregulated in adult sea lice exposed to azamethiphos (Table 3). Also, increased expression was observed for both females and males in contigs annotated as members of the ABCA/D subfamilies in response to azamethiphos (Figure 4). An opposite effect was observed in contig 1285 annotated as an ABCF transporter, which was downregulated in sea lice exposed to azamethiphos. Furthermore, different transcriptomic responses were observed between sexes in response to azamethiphos, where the ABCB and ABCC subfamilies were only up-regulated in males. In response to deltamethrin, the ABCD/E/F subfamilies were highly regulated in adult sea lice (Table 3). Expression profiles of ABC transporters between females and males were similar (Figure 5). However, the contig 4421 annotated for ABCB transporter was upregulated in males exposed to deltamethrin, while females presented a high expression of contig 3679 annotated as a member of the ABCF subfamily.Additionally, in order to validate the transcription profiles obtained from sequencing data analysis, RT-qPCR was conducted on one represent of ABC transporters subfamily. The Pearson correlation evidenced a high linear dependence of fold change values obtained from both RNA-Seq and RT-qPCR for each ABC transporters in sea louse exposed to azamethiphos and deltamethrin (Figure 6).

**Table 3:** **Differential transcripts expression for ABC proteins subfamily en adults exposed to delousing drugs and control groups**

		**Deltamethrin vs Control**		**Azamethiphos vs Control**	
**Feature ID**	**ABC protein subfamily**	**Fold change (Log2)**	**P-value**	**Fold change (Log2)**	**P-value**
contig161	ABCA	−1.888	0	1.390	0
contig17121	ABCA	1.364	0	−1.696	0
contig44438	ABCA	0.000	0	0.000	0
contig53840	ABCA	1.000	1	1.000	1
contig57423	ABCA	1.000	1	1.000	1
contig57865	ABCA	1.000	1	1.000	1
contig58749	ABCA	1.000	1	1.000	1
contig4521	ABCB	1.637	0	−2.799	0
contig51710	ABCB	0.000	0	0.000	0
contig53281	ABCB	0.000	1.83E-13	0.000	2.36E-13
contig53886	ABCB	−3.065	0	3.943	0
contig55404	ABCB	1.000	1	0.000	0
contig55589	ABCB	1.000	1	1.000	1
contig56255	ABCB	1.000	1	1.000	1
contig57826	ABCB	1.000	1	1.000	1
contig5805	ABCB	1.107	0	−3.025	0
contig60252	ABCB	1.000	1	1.000	1
contig62765	ABCB	1.000	1	1.000	1
contig9296	ABCB	−1.344	0	−11.758	0
contig13883	ABCC	−2.880	0	−8.558	0
contig14757	ABCC	−2.056	0	1.172	0
contig17496	ABCC	−2.317	0	2.817	0
contig2074	ABCC	−1.473	0	4.793	0
contig25186	ABCC	−1.553	0	−4.945	0
contig2865	ABCC	−1.403	0	−5.229	0
contig3447	ABCC	−1.169	0	−4.882	0
contig46370	ABCC	0.000	0	−2.778	0
contig51756	ABCC	0.000	0	0.000	0
contig53821	ABCC	−1.792	0	0.000	0
contig6109	ABCC	−1.346	0	−4.620	0
contig7999	ABCC	1.043	2.45E-05	−3.440	0
contig9208	ABCC	−1.109	0	−5.464	0
contig9779	ABCC	−2.604	0	−1.776	0
contig9824	ABCC	−1.307	0	−6.285	0
contig13006	ABCD	1.394	0	−1.665	0
contig3071	ABCD	−1.338	0	−4.260	0
contig44400	ABCD	3.125	0	155.119	0
contig45476	ABCD	0.000	0	0.000	0
contig3516	ABCE	1.215	0	−2.187	0
contig389	ABCE	1.320	0	−3.150	0
contig4979	ABCE	1.233	0	−2.241	0
contig6402	ABCE	1.890	0	−2.037	0
contig6459	ABCE	1.159	0	−3.435	0
contig1285	ABCF	1.226	0	−3.338	0
contig3679	ABCF	1.577	0	−2.707	0
contig389	ABCF	1.359	0	−3.163	0
contig41019	ABCF	0.000	0	0.000	0
contig51772	ABCF	0.000	0	6.135	0
contig51868	ABCF	8.776	0	−1.255	3.85E-08
contig56842	ABCF	1.000	1	1.000	1
contig57870	ABCF	1.000	1	1.000	1
contig15272	ABCG	2.250	0	1.016	0.781
contig52434	ABCG	0.000	0	0.000	0
contig59723	ABCG	0.000	0	0.000	0
contig1008	ABCH	1.182	0	−3.219	0
contig4979	ABCH	1.252	0	−2.339	0
contig6402	ABCH	1.715	0	−2.137	0

### Single nucleotide polymorphism (SNP) mining

A total of 52 SNPs were identified in 17 contigs that annotated for ABC transporter sequences in *C. rogercresseyi* (Additional file 1: Table S3). For contigs annotated for the ABCA subfamily, a non-synonymous variation was identified in the TMD2 domain (Additional file 1: Table S3). Three contigs presented SNPs variations for the ABCB subfamily, one of which, contig 9296, had a non-synonymous variation in the NBD1 domain (Additional file 1: Table S3). For the ABCC subfamily, seven contigs presented SNPs in the open reading frame and 3′UTR regions (Additional file 1: Table S3). In ABCD/E/G/H subfamilies, the identified variations were synonymous or were present in the 3′UTR region (Additional file 1: Table S3). Nevertheless, more studies are necessary to determinate the function of non-synonymous variations in ABC transporters and the possible association of these with delousing drug resistance.

## Discussion

The ABC family is composed of proteins that include a wide range of compounds both within and exterior to the cell. In eukaryotes, many of the functions that these proteins have in the cell remain unknown. However, in *Drosophila melanogaster* these proteins have been observed to participate in the modulation of molting hormones such as the ecdysteroids. These transporters have also been found in insects to play a role in insecticide tolerance [16,40]. Currently in arthropods, 64, 56, and 46 ABC proteins have been identified in *D. pulex* [12], *Drosophila* [16], and *T. japonicus* [23], respectively. The present study found 57 contigs with homology to the different ABC proteins subfamilies during distinct larval and adult stages of *C. rogercresseyi* development [35]. Of these contigs, members belonging to eight of the ABC subfamilies (A-H) described in arthropods were identified [16].

Differences in the expression of the eight ABC proteins subfamilies were evaluated *in silico* during the distinct developmental stages of *C. rogercresseyi*. The ABCA subfamily was upregulated during the nauplius I-II and chalimus III-IV larval stages. The physiological function of this transporter subfamily is still unclear in arthropod organisms [41]. However, in *T. castaneium*, blocking this subfamily causes mortality in pupae and adults [21]. Taking the results obtained in *T. castaneum* together with the greater expression levels observed in juvenile *C. rogercresseyi*, it is possible to suggest that the ABCA subfamily plays a role during early ontogenetic development.

In turn, the ABCB subfamily has been widely studied in humans due to its function as a MRP in studies related to the control of cancer [42,43]. Moreover, this subfamily has been implicated in the molting process and the developmental transition from pupa to adult in *T. castaneum* [21]. The present study found greater expression levels in juvenile chalimus III-IV stages and in adult *C. rogercresseyi*, which is a result similar to that observed in *T. castaneum* [21] and *T. japonicus* [23]. Taken together, these findings indicate greater metabolic activity in response to pharmaceutical treatments during the parasitic stages of *C. rogercresseyi*.

Similar to the ABCB subfamily, ABCC transporters have detoxifying activities and present specific binding sites to drugs (MRPs) [13]. In *C. rogercresseyi*, transporters associated with ABCC1 and ABCC4 were identified and overexpressed throughout the course of the nauplius I-II larval stages. The ABCC1 transporters are considered “long” MRPs and present an extra transmembrane domain in the N-terminal termed TMD_0_. This is compared to “short” MRPs, among which is ABCC4 [44]. Long MRPs, or MRP1, have been associated with xenobiotic resistance in humans [45], *T.ni* [28], and *L. salmonis* [31]. Other ABC protein member of this subfamily is the sulfonylurea receptor (SUR). In arthropod SUR proteins have been related to chitin synthesis and in some reports suggest that SUR are putative targets for some insecticide [16,46]. For *C. rogercresseyi* some contigs with high homology with SUR gene were highly regulated in larval stages, which can be related to the sea lice cuticle biogenesis. On the other hand, the high transcript levels observed during the larval stages of *C. rogercresseyi* allow for associating the C subfamily, in addition to its detoxifying characteristics, to processes related to development and maturation during early developmental stages, as has been observed in other invertebrates [16,47].

The ABCD subfamily in *C. rogercresseyi* was overexpressed in the nauplius I-II and chalimus I-II larval stages. In contrast to other transporter types, this subfamily is found in membrane-bound peroxisome, and its function is to transport fatty acids to the interior of this organelle [16,41,48]. These transporters have also been observed to play a role in the metabolism and development of arthropods [49]. Congruent with that observed for *C. rogercresseyi*, this transporter subfamily has been found expressed during all of the developmental stages in *T. japonicus* [23].

From the infective copepodid stages, an increased expression was observed for the ABCE and ABCF subfamilies in *C. rogercresseyi.* In humans, the ABCE1 transporter has inhibitory actions on RNase L, an important enzyme related to interferon functions in response to the presence of a virus [48]. It has also been found that blocking the ABCE-F transporters in *T. castaneum* causes mortality in the pre-pupa stage, thereby impeding development into adults [21]. Moreover, the ABCE transporter presents ubiquitous expression during all developmental stages in *T. japonicus* [23].

The ABCG subfamily of transporters corresponds to the haft transporter and presents inverted domains in the extreme C-terminal [12]. In *C. rogercresseyi*, this subfamily was principally overexpressed during the nauplius II larval stage. The ABCG transporter is homologous to the white protein that forms heterodimers in *D. melanogaster* with the brown and scarlet proteins, which themselves act as a precursor to pigmentation [50]. In *T. castaneum*, the inactivation of this gene provoked an arrest of development during the pre-pupa stages [21]. The ABCG transporters have also been found to induce the expression of genes linked to the molting hormone 20-ecdysone (20E) in *D. melanogaster* [51]. Given the expression of this transporter during the nauplius II stage in *C. rogercresseyi,* it could be associated with development towards the copepodid stage.

The final subfamily identified was the ABCH subfamily, which presents a domain formation similar to that of the ABCG subfamily [12]. In *C. rogercresseyi*, three contigs were identified that annotated for the ABCH subfamily, and these were overregulated in the nauplius II stage. Despite that the function of the ABCH transporters is still unclear, in *T. cantaneum* these have been found to play a fundamental role in the transport of lipids to the cuticle, thereby generating a hydrophobic barrier for the organism [21]. On the other hand, in *T. urticae* these types of transporters could be involved in the process of diapause [52].

The ABC transporters have been widely studied for their role as MRPs, especially the ABCB and ABCC subfamilies. The ABC transporters have been found to participate in the detoxification of pyrethroids and avermectins, among other chemicals generally used in invertebrate pest control [53-55]. For example, increased transcript levels of the ABCB1 (P-gp) transporter in *L. salmonis* has been linked to generated resistance to EMB [10,11]. Similarly, an increased gene expression of ABC transporters in *A. aegypti* populations resistant to pyrethroids suggests the participation of these transporters in detoxifying processes [29]. In addition to pyrethroids, the effect of organophosphates on expression levels of ABC transporters has been evaluated in rats, showing that exposure to diazinon induced an increased intestinal expression of *P-gp* [56]. Moreover, in resistant strains of *Rhipicephalus microplus* treated with an inhibitor of ABCB transporters and exposed to different concentrations of chlorpyrifos, the lethal concentration of this organophosphate was reduced as compared to strains not treated with an inhibitor. These findings reveal the interaction between ABC transports and the detoxification process of organophosphates [53].

The present study evaluated the transcriptomic response of the ABC transporters identified in adult *C. rogercresseyi* individuals exposed to the pyrethroid deltamethrin and the organophosphate azamethiphos. For both treatments, the ABCB and ABCC transporter subfamilies presented higher expression levels in salmon lice exposed to the pharmaceuticals. Additionally, differences in expression profiles were observed between males and females, a result which has also been obtained in EBM resistant strains of *L. salmonis* [57]. In regards to the ABCG subfamily, these have been found to transport drugs and sterols [16], and in *P. xylostella* resistant to insecticides, an increased expression of the ABCH transporters has been reported, thus suggesting a possible detoxifying role [58]. However, in *C. rogercresseyi*, no significant change in transcript levels of these two subfamilies was found in adults exposed to deltamethrin or azamethiphos.

The presence of mutations in genes coding for the ABC transporters can generate changes in molecular functions and related biological processes. For example, mutations in human ABCD transporters have been found to cause adrenal insufficiency and the demyelination of neurons [59]. Moreover, variations in transporters linked to processes of detoxification can induce resistance in organisms, such as with the insertion of tyrosine in the ABCC4 transporter of *Bombyx mori*, where strains with this insertion were resistant to the toxin Cry1Ab [60]. Another possible mutation is through SNP variations, which could be an important tool in identifying organisms resistant or susceptible to certain drugs. For example, 13 polymorphisms have been identified in humans in distinct ABC transporter subfamilies, and these have led to associations between individual responses and distinct therapeutic drug treatments [61]. Apart from this, the nematode *Onchocerca volvulus* presents alterations in P-gp transporter functions in association with the presence of SNPs that generate non-synonymous changes [62]. The present study identified 17 ABC transporters related to polymorphisms in a single nucleotide. The identified SNPs evidenced both synonymous and non-synonymous mutations in conserved domains and UTR regions. Future studies will focus on relating the reported mutations with the response of *C. rogercresseyi* to the distinct pharmaceuticals used in infestation control.

## Conclusions

The present study applied genomic approaches to identify 57 sequences annotating for the eight members of the ABC transporter subfamilies in *C. rogercresseyi*. The ABC transporters were evaluated *in silico* and demonstrated changes in expression throughout the developmental stages of the salmon louse. Additionally, the expression profiles of ABC transporters in adult individuals exposed to deltamethrin or azamethiphos were evaluated, finding increased expression levels for the ABCB and ABCC subfamilies. Finally, mutations were found in 17 ABC transporters, with these being located in both the open reading frame and UTR regions. Future studies will evaluate the effects that the identified SNPs have in *C. rogercresseyi* strains resistant or susceptible to the drugs used for its control in the Chilean salmon aquaculture industry.

## Additional file

Additional file 1: Table S1. Primer list for ABC transporters identified in *C. rogercresseyi* for qPCR validation. **Table S2.** BLASTx analysis of 57 *C. rogercresseyi* ABC proteins. **Table S3.** List of SNPs identified for ABC proteins from *C. rogercresseyi.*
