# Supplementary material for: Transcriptomic insights on the ABC transporter gene family in the salmon louse Caligus rogercresseyi
Source: Parasit Vectors. 2015 Apr 9;8:209. doi: 10.1186/s13071-015-0801-x (PMC4403882; doi:10.1186/s13071-015-0801-x)
Supplement: Additional file 1: Table S1. — Primer list for ABC transporters identified in C. rogercresseyi for qPCR validation. Table S2. BLASTx analysis of 57 C. rogercresseyi ABC proteins. Table S3. List of SNPs identified for ABC proteins from C. rogercresseyi. [file 13071_2015_801_MOESM1_ESM.docx]

**Table S1.** Primer list for ABC transporters identified in *C. rogercresseyi* for qPCR validation

| **Contig** | **Gene** | **Primer Name** | **Primer Sequence** |
| --- | --- | --- | --- |
| Contig 23495 | β-tubulin | Cr_β-tubulin_2F | TTTGTTGTGTGAGCTCTGGG |
|  |  | Cr_β-tubulin_2R | GCTGATCTCCGAAAACTTGC |
| Contig 616 | ABCA | Cr_ABCA_F2 | CCAAGGGAAGCCTGGAGAAG |
|  |  | Cr_ABCA_R2 | GGGATTGATGACCACACCGT |
| Contig 4521 | ABCB | CrABCB XII_F | GAGTCCCGGCGTGCGTCCTCAGT |
|  |  | CrABCB XII_R | GGCCTCTGTGGTCATGGGGGCCT |
| Contig 9208 | ABCC | CrABCC XIII_F | TGTCAGTGGATGTCTCTCGGATCGCA |
|  |  | CrABCC XIII_R | TGCCTCCAATGATGGCCCAGCCCAG |
| Contig 3071 | ABCD | Cr_ABCD_F | CCGGCACAACTTCAAGAGGA |
|  |  | Cr_ABCD_R | AGGGAGTGTCCAATTGAGCG |
| Contig 3561 | ABCE | Cr_ABCE_F | TCTGCGTCAAGAAGTGTCCC |
|  |  | Cr_ABCE_R | GGAGCTTGAAGGAGTTGGCT |
| Contig 1258 | ABCF | Cr_ABCF_F2 | GATGTCCAGTCCCCAAGGTC |
|  |  | Cr_ABCF_R2 | TTCCTCTCGTCACTGGGCTA |
| Contig 15272 | ABCG | Cr_ABCG_F2 | GCACTAATCCACGACCCAGA |
|  |  | Cr_ABCG_R2 | TGCGTGGTGATGAGAATGGT |
| Contig 6402 | ABCH | Cr_ABCH_F | AGAAAGACCCCAGGGCTACT |
|  |  | Cr_ABCH_R | CCCAGGGACCCCTCTTTCTA |

**Table S2.** BLASTx analysis of 57 *C. rogercresseyi* ABC proteins

| **Query** | **Lowest (E-value)** | **Lenght Predict Amino Acid Sequence** | **Accession**  **(E-value)** | **Description (E-value)** |
| --- | --- | --- | --- | --- |
|  |  |  |  |  |
| ***ABCA*** |  |  |  |  |
| contig161 | 0.00 | 2,208 | EFX87570 | ABC protein, subfamily ABCA [Daphnia pulex]. |
| contig58749 | 2.72E-45 | 192 | EFX87571 | ABC protein, subfamily ABCA [Daphnia pulex]. |
| contig17121 | 1.21E-14 | 268 | EFX87570 | ABC protein, subfamily ABCA [Daphnia pulex]. |
| contig44438 | 2.53E-14 | 109 | EFX89859 | ABC protein, subfamily ABCA [Daphnia pulex]. |
| contig57423 | 3.04E-13 | 193 | EFX87570 | ABC protein, subfamily ABCA [Daphnia pulex]. |
| contig53840 | 4.30E-09 | 120 | EFX89859 | ABC protein, subfamily ABCA [Daphnia pulex]. |
| contig57865 | 1.01E-07 | 159 | EFX87570 | ABC protein, subfamily ABCA [Daphnia pulex]. |
| ***ABCB*** |  |  |  |  |
| contig4521 | 0,00 | 2,064 | EFX85237 | ABC transporter, subfamily ABCB/MDR [Daphnia pulex]. |
| contig5805 | 1.06E-90 | 775 | EFX85237 | ABC transporter, subfamily ABCB/MDR [Daphnia pulex]. |
| contig62765 | 1.07E-51 | 155 | EFX86431 | ABC protein, subfamily ABCB/MDR [Daphnia pulex]. |
| contig60252 | 1.28E-30 | 171 | EFX85237 | ABC transporter, subfamily ABCB/MDR [Daphnia pulex]. |
| contig56255 | 3.90E-27 | 193 | EFX86431 | ABC protein, subfamily ABCB/MDR [Daphnia pulex]. |
| contig9296 | 1.88E-24 | 137 | EFX85237 | ABC transporter, subfamily ABCB/MDR [Daphnia pulex]. |
| contig55589 | 4.06E-17 | 192 | EFX86431 | ABC protein, subfamily ABCB/MDR [Daphnia pulex]. |
| contig57826 | 2.79E-15 | 270 | EFX85237 | ABC transporter, subfamily ABCB/MDR [Daphnia pulex]. |
| contig53281 | 8.94E-10 | 215 | EFX86431 | ABC protein, subfamily ABCB/MDR [Daphnia pulex]. |
| contig51710 | 1,61E-09 | 163 | EFX86431 | ABC protein, subfamily ABCB/MDR [Daphnia pulex]. |
| contig55404 | 3.28E-05 | 108 | EFX86431 | ABC protein, subfamily ABCB/MDR [Daphnia pulex]. |
| contig53886 | 5.73E-07 | 120 | EFX85237 | ABC protein, subfamily ABCB/MDR [Daphnia pulex]. |
| ***ABCC*** |  |  |  |  |
| contig2074 | 0.00 | 1,438 | EFX72656 | ABC protein, subfamily ABCC [Daphnia pulex]. |
| contig9779 | 0.00 | 798 | EFX68457 | ATP-binding cassette, sub-family C, Member 4 [Daphnia pulex]. |
| contig9824 | 7.79E-112 | 1,417 | EFX72657 | ABC protein, subfamily ABCC [Daphnia pulex]. |
| contig7999 | 2.34E-168 | 1,778 | EFX82733 | ABC protein, subfamily ABCC [Daphnia pulex]. |
| contig6109 | 2.42E-168 | 1,978 | EFX72656 | ABC protein, subfamily ABCC [Daphnia pulex]. |
| contig3447 | 1.22E-59 | 226 | EFX68457 | ATP-binding cassette, sub-family C, Member 4 [Daphnia pulex]. |
| contig14757 | 2.90E-73 | 569 | EFX72656 | ABC protein, subfamily ABCC [Daphnia pulex]. |
| contig25186 | 2.93E-64 | 281 | EFX68457 | ATP-binding cassette, sub-family C, Member 4 [Daphnia pulex]. |
| contig51756 | 2.67E-43 | 132 | EFX72656 | ABC protein, subfamily ABCC [Daphnia pulex]. |
| contig46370 | 1.99E-37 | 208 | EFX72656 | ABC protein, subfamily ABCC [Daphnia pulex]. |
| contig13883 | 1.01E-18 | 174 | EFX68457 | ATP-binding cassette, sub-family C, Member 4 [Daphnia pulex]. |
| contig17496 | 8,51E-09 | 150 | EFX82733 | ABC protein, subfamily ABCC [Daphnia pulex]. |
| contig53821 | 2.01E-25 | 210 | EFX86431 | ABC protein, subfamily ABCC [Daphnia pulex]. |
| contig2865 | 6.11E-142 | 1,722 | EFX68442 | sulfonylurea receptor 2B-like protein [Daphnia pulex]. |
| contig9208 | 3.39E-144 | 1,507 | EFX68442 | sulfonylurea receptor 2B-like protein [Daphnia pulex]. |
| ***ABCD*** |  |  |  |  |
| contig13006 | 4.00E-120 | 591 | EFX65057 | ABC protein, subfamily ABCD [Daphnia pulex]. |
| contig3071 | 1.98E-105 | 598 | EFX65057 | ABC protein, subfamily ABCD [Daphnia pulex]. |
| contig45476 | 1.47E-67 | 159 | EFX83241 | ABC transporter, subfamily D [Daphnia pulex]. |
| contig44400 | 9.73E-09 | 273 | EFX65057 | ABC protein, subfamily ABCD [Daphnia pulex]. |
| ***ABCE*** |  |  |  |  |
| contig3516 | 0.00 | 1,744 | AAZ75681 | ABC transporter, ABCE1 [Sarcoptes scabiei]. |
| contig6469 | 5.60E-12 | 1,326 | XP_003690922 | ATP-binding cassette, sub-family E, Member 1 [Apis florea]. |
| contig4979 | 7.25E-12 | 900 | XP_005105420 | ATP-binding cassette, sub-family E [Musca domestica]. |
| contig389 | 4.50E-11 |  | XP_002121964 | ATP-binding cassette, sub-family E [Musca domestica]. |
| contig6402 | 7.16E-10 | 1,045 | XP_003690922 | ATP-binding cassette, sub-family E, Member 1 [Apis florea]. |
| ***ABCF*** |  |  |  |  |
| contig3679 | 0.00 | 1,272 | EFX69544 | ABC protein, subfamily ABCF [Daphnia pulex]. |
| contig389 | 0.00 | 135 | EFX73813 | ABC protein, subfamily ABCF [Daphnia pulex]. |
| contig1285 | 0.00 | 634 | EFX88800 | ABC protein, subfamily ABCF [Daphnia pulex]. |
| contig41019 | 2.10E-32 | 104 | EFX69544 | ABC protein, subfamily ABCF [Daphnia pulex]. |
| contig57870 | 6.20E-17 | 112 | EFX73813 | ABC protein, subfamily ABCF [Daphnia pulex]. |
| contig56842 | 1.75E-15 | 165 | EFX73813 | ABC protein, subfamily ABCF [Daphnia pulex]. |
| contig51772 | 9.58E-08 | 90 | EFX69544 | ABC protein, subfamily ABCF [Daphnia pulex]. |
| contig51868 | 5.53E-06 | 192 | EFX69544 | ABC protein, subfamily ABCF [Daphnia pulex]. |
| ***ABCG*** |  |  |  |  |
| contig15272 | 0.00 | 1,171 | EFX69628 | ABC protein, subfamily ABCG [Daphnia pulex]. |
| contig59723 | 3.05E-13 | 117 | EFX85518 | ABC protein, subfamily ABCG [Daphnia pulex]. |
| contig52434 | 9.62E-09 | 148 | EFX68572 | ABC protein, subfamily ABCG [Daphnia pulex]. |
| ***ABCH*** |  |  |  |  |
| contig4979 | 0.00 | 900 | EFX78467 | ABC protein, subfamily ABCH [Daphnia pulex]. |
| contig6402 | 0.00 | 1,045 | EFX78468 | ABC protein, subfamily ABCH [Daphnia pulex]. |
| contig1008 | 2.63E-58 | 939 | EFX71371 | ABC protein, subfamily ABCH [Daphnia pulex]. |

**Table S3.** List of SNPs identified for ABC proteins from *C. rogercresseyi*

| **Mapping** | **Reference Position** | **Allele** | **Frequency** | **Substitution** | **Domain** |
| --- | --- | --- | --- | --- | --- |
| ABCA_contig161 | 49 | T/C | 43.75 | synonymous | TMD1 |
| ABCA_contig161 | 349 | T/C | 48.57 | synonymous | TMD1 |
| ABCA_contig161 | 1948 | C/T | 36.11 | synonymous | TMD2 |
| ABCA_contig161 | 4696 | A/T | 40.50 | synonymous | TMD2 |
| ABCA_contig161 | 5792 | G/A | 36.80 | non-synonymous | TMD2 |
| ABCA_contig161 | 5938 | G/A | 39.28 | synonymous | NBD2 |
| ABCA_contig161 | 6683 | T/C | 42.30 | 3'UTR |  |
| ABCB_contig4521 | 4225 | T/C | 40.35 | synonymous | NBD2 |
| ABCB_contig4521 | 3943 | C/G | 35.84 | synonymous | NBD2 |
| ABCB_contig4521 | 3880 | C/T | 39.02 | synonymous | NBD2 |
| ABCB_contig4521 | 2473 | T/C | 43.18 | synonymous | NBD2 |
| ABCB_contig5805 | 292 | G/A | 41.73 | synonymous | TMD1 |
| ABCB_contig5805 | 650 | T/C | 56.73 | synonymous | NBD2 |
| ABCB_contig5805 | 1378 | A/T | 52.55 | synonymous | NBD2 |
| ABCB_contig9296 | 35 | T/C | 40.38 | non-synonymous | NBD1 |
| ABCC_contig2074 | 2560 | C/A | 40 | non-synonymous | TMD2 |
| ABCC_contig2074 | 1817 | A/G | 35.29 | synonymous | NBD1 |
| ABCC_contig2074 | 534 | A/G | 39.72 | non-synonymous | TMD1 |
| ABCC_contig2865 | 22 | A/G | 97.58 | 5'UTR |  |
| ABCC_contig2865 | 157 | G/A | 44.79 | 5'UTR |  |
| ABCC_contig2865 | 3534 | C/A | 37.5 | synonymous | TMD1 |
| ABCC_contig9208 | 237 | T/A | 52.77 | non-synonymous | NH2 |
| ABCC_contig9208 | 627 | A/G | 55.26 | non-synonymous | NH2 |
| ABCC_contig9208 | 2150 | C/A | 44.64 | synonymous | NBD1 |
| ABCC_contig9208 | 2501 | T/C | 43.13 | synonymous | - |
| ABCC_contig9208 | 2661 | A/G | 41.79 | non-synonymous | TMD2 |
| ABCC_contig9208 | 3854 | T/C | 41.66 | synonymous | NBD2 |
| ABCC_contig9779 | 522 | T/G | 60 | non-synonymous | TMD1 |
| ABCC_contig9824 | 4136 | A/T | 77.77 | 3'UTR |  |
| ABCC_contig9824 | 4089 | A/C | 63.76 | 3'UTR |  |
| ABCC_contig9824 | 3928 | T/C | 35.13 | 3'UTR |  |
| ABCC_contig9824 | 3759 | A/T | 39.83 | 3'UTR |  |
| ABCC_contig9824 | 3541 | A/T | 38.46 | 3'UTR |  |
| ABCC_contig9824 | 2857 | A/G | 50 | synonymous | NBD2 |
| ABCC_contig9824 | 2488 | T/C | 41.37 | synonymous | NBD2 |
| ABCC_contig9824 | 1078 | C/T | 42.10 | synonymous | - |
| ABCC_contig9824 | 763 | T/C | 47.82 | synonymous | NBD1 |
| ABCC_contig6109 | 1078 | C/T | 62.96 | non-synonymous | TMD1 |
| ABCC_contig6109 | 1150 | G/A | 53.96 | non-synonymous | TMD1 |
| ABCC_contig6109 | 2391 | T/C | 39.06 | synonymous | TMD2 |
| ABCC_contig6109 | 3872 | C/G | 52.08 | 3'UTR |  |
| ABCC_contig6109 | 4107 | A/C | 46.99 | 3'UTR |  |
| ABCC_contig6109 | 4155 | C/T | 36.29 | 3'UTR |  |
| ABCC_contig6109 | 4176 | A/C | 41.01 | 3'UTR |  |
| ABCC_contig6109 | 4181 | C/T | 40.99 | 3'UTR |  |
| ABCC_contig6109 | 4549 | C/T | 42.06 | 3'UTR |  |
| ABCC_contig6109 | 4652 | T/C | 45.33 | 3'UTR |  |
| ABCC_contig6109 | 4689 | A/C | 40.84 | 3'UTR |  |
| ABCD_contig13006 | 1253 | A/T | 39.13 | synonymous | NBD |
| ABCD_contig13006 | 948 | G/T | 48.38 | non-synonymous | - |
| ABCD_contig13006 | 668 | C/T | 37.5 | synonymous | TMD1 |
| ABCE_contig6459 | 169 | C/A | 35.57 | 5'UTR |  |
| ABCE_contig6459 | 284 | C/G | 36.60 | synonymous | NH2 |
| ABCE_contig6459 | 638 | C/T | 51.24 | synonymous | NH2 |
| ABCE_contig6459 | 1127 | T/C | 52.78 | synonymous | NDB1 |
| ABCE_contig6459 | 1166 | A/T | 52 | 3'UTR |  |
| ABCE_contig6459 | 2401 | C/A | 39.61 | 3'UTR |  |
| ABCE_contig6459 | 3152 | C/T | 58.68 | 3'UTR |  |
| ABCE_contig6459 | 3256 | G/A | 38.86 | 3'UTR |  |
| ABCE_contig6459 | 3338 | G/A | 44.8 | 3'UTR |  |
| ABCE_contig6459 | 3422 | C/T | 37.17 | 3'UTR |  |
| ABCE_contig6459 | 3470 | C/G | 41.79 | 3'UTR |  |
| ABCE_contig6459 | 3512 | A/G | 36.36 | 3'UTR |  |
| ABCF_contig1285 | 1731 | A/G | 57.60 | synonymous | TMD2 |
| ABCF_contig1285 | 1716 | T/C | 51.07 | synonymous | TMD2 |
| ABCF_contig1285 | 1194 | A/G | 38.33 | synonymous | TMD2 |
| ABCF_contig3679 | 3693 | G/A | 39.71 | synonymous | NBD1 |
| ABCF_contig3679 | 2874 | C/A | 35.71 | synonymous | NBD1 |
| ABCF_contig3679 | 2616 | G/T | 59.89 | synonymous | NBD1 |
| ABCF_contig3679 | 2538 | A/C | 41.57 | synonymous | NBD1 |
| ABCF_contig3679 | 2082 | G/A | 35.07 | synonymous | NBD1 |
| ABCF_contig3679 | 2037 | T/C | 44.69 | synonymous | NBD1 |
| ABCG_contig15272 | 3476 | G/A | 45.45 | 3'UTR |  |
| ABCG_contig15272 | 3332 | C/A | 61.53 | 3'UTR |  |
| ABCG_contig15272 | 2378 | A/G | 42.85 | synonymous | NBD1 |
| ABCG_contig15272 | 1571 | G/A | 45.45 | synonymous | NBD1 |
| ABCG_contig15272 | 1553 | C/T | 36.36 | synonymous | NBD1 |
| ABCG_contig15272 | 1025 | C/T | 53.84 | synonymous | - |
| ABCG_contig15272 | 1001 | C/A | 45.45 | synonymous | - |
| ABCG_contig15272 | 920 | A/G | 50 | synonymous | TMD2 |
| ABCG_contig15272 | 641 | A/G | 50 | synonymous | TMD2 |
| ABCG_contig15272 | 582 | C/A | 45.45 | synonymous | TMD2 |
| ABCG_contig15272 | 297 | C/A | 54.54 | synonymous | TMD2 |
| ABCH_contig1008 | 2793 | G/C | 67.71 | 3'UTR |  |
| ABCH_contig1008 | 2724 | C/A | 51.80 | 3'UTR |  |
| ABCH_contig1008 | 2698 | T/C | 57.72 | 3'UTR |  |
| ABCH_contig1008 | 2653 | C/T | 42.99 | 3'UTR |  |
| ABCH_contig1008 | 2642 | T/G | 58.53 | 3'UTR |  |
| ABCH_contig1008 | 2640 | T/C | 73.65 | 3'UTR |  |
| ABCH_contig1008 | 2637 | T/C | 62.18 | 3'UTR |  |
